# Progress in Surgery

**Published:** 1900-06-30

**Authors:** 


### Progress in Surgery.

### CANCER.

**Etiology.**—Sir William Banks[1] suggests, as an impression left by an experience of 33 years, that the undoubted increase in the prevalence of cancer may be associated with the larger amounts of food ingested now than formerly. He has noticed that the cancer patient is well-nourished, as a rule, the spare and emaciated being quite the exception. He criticises the statement that "vegetarians and rice-eaters" are as subject to the disease as "flesh-eaters or gross feeders." He also holds that chronic interstitial mastitis is more frequently than is supposed the forerunner of carcinoma, and advises that such breasts should be more frequently removed as a preventive measure.

**Infection.**—Bland Sutton[2] points out the importance of the venous system as a channel whereby cancer cells are frequently transmitted as emboli, in addition to their usual route, the lymphatics, and apart from the entrance into the venous system of cells traversing the thoracic duct. The minute lymphatic and venous channels cut, or infected glands compressed during removal, are both fruitful sources of infection to the peritoneum or subcutaneous tissue, &c. This should be remembered in operating, so that not only should the growth not be cut into, but the efferent channels and neighbouring glands preserved intact. Shattock[3] cites an instance of a rabbit, not the subject of experiment, suffering from "highly-marked carcinoma" in both uteri, multiple and extensive tumours revealing a columnar-celled type, with disseminated growths in the peritoneum. This he quotes as bearing on Dr. Lack's experiment of August, 1899, whereby carcinomatous foci were said to be induced in the peritoneal cavity, and secondarily in the thorax of a rabbit, by allowing scrapings of cut sections of ovary to escape into the abdominal cavity. Quoting Lubarsch, he mentions the recent experiments where emulsions of various glands (omitting the ovary, though including the testicle) were injected into the venous system of rabbits without producing any results. He raises the question of a possible immunity acquired by an individual receiving successive implantations of epithelium. Warts have been inoculated on dogs, and they have died away; successive attempts at inoculation have failed. He instances those cases where, after some slight injury to the palm of the hand, epidermal cysts have grown beneath the corium, with walls having every resemblance to skin, and yet showing no tendency to form carcinoma. Similarly the rupture of ovarian dermoids are followed by the formation of multiple cysts, but no carcinoma.

**Treatment.**—Coley[4] reports the complete cure of a recurrent inoperable spindle-celled sarcoma of the parotid gland, with secondary growth in the submaxillary gland, by the extensive use of the "mixed toxins." The toxins were injected into the tumour, which measured $3\frac{1}{2}$ in. by 4 in. No recurrence after two and a half years. Wyeth supposed that the injections may have caused inflammation, and that this might have been the cause of the improvement. A. R. Robinson[5] holds that cases of cutaneous cancer, especially those about the face, are better removed by means of caustics, such as arsenious acid, than by means of the knife. He holds that less deformity results upon the judicious use of caustics, and when there is as yet no glandular infection, and the position is safe for its use, the action of this caustic is more far-reaching and complete than would be a more extensive use of the knife. For, he says, the acid, destroying as it does the pathological epithelium much more readily than the normal tissues, destroys the outlying plugs and processes, which may be missed by the knife. The method he uses is, briefly, the removal of the superficial growth by means of the curette or possibly the knife, and then the application of equal parts of arsenious acid, gum acacia, mixed into a "butter" with water. He quotes cases of cure, one ten years ago, with practically no scar and no recurrence. The after treatment is a simple antiseptic ointment. A little suppuration will encourage the granulation tissue to fill up the cavity and leave less scar. G. Betton Massey[6] publishes more cases of malignant diseases treated by the "massive cataphoric diffusion of the nascent electrolytic salts of mercury," using direct currents of 200-1,200 milliampères through gold anodes coated with metallic mercury. He records four operable and six inoperable cases cured out of 39 treated; seven others are yet uncertain; 22 were failures—two of the latter died under treatment (one a child with sarcoma of orbit, the other an epithelioma (recurrent) of tonsil); nine of them had secondary growths latent which developed subsequently, while there was no recurrence at primary seat. The remaining 13 he attributed to insufficient current being possible on account of the (*a*) proximity to the brain; (*b*) want of an anæsthetic; (*c*) failure of batteries to give strong enough current in the early applications.

**Experiments.**—Bra and Mongour[7] have injected "Nectrianin," the principle of the Nectria Ditissima parasite of the cancer of trees, into animals and human beings. There was no appreciable effect on healthy animals, but in the cancerous subject the injection of doses of 5 c.c. several times a week was followed by chills, accelerated pulse, thirst, and headache. After several hours a crisis occurred with perspiration, polyuria, and profound sleep. It is claimed that hæmorrhage is diminished, fœtid discharges suppressed, and a tendency noted of the tumour to become covered with epidermis. It is suggested that this treatment may be used coincidently with surgical measures.

At the State Cancer Laboratory[8] (Buffalo) infected lymph nodes containing "Russell's bodies" had been dried and the powder injected into animals. These died three or four weeks afterwards, showing lymph nodes containing large numbers of "Russell's bodies."

**Interesting Cases.** — A. L. Miller[9] publishes and describes a very rare instance of what seems to be primary sarcoma of the pericardium of a boy of 13. It was of the small-round-celled variety; the peribronchial and mediastinal glands were very slightly enlarged, and no other glands affected. The epicardium was free, while the pericardium was enormously thickened. The tumour containing the heart with the lungs together weighed $6\frac{1}{4}$ lbs. The symptoms were chiefly dyspnœa, and towards the end dropsy. There were no cardiac murmurs, and the impulse was markedly increased.

E. T. Fison[10] quotes an interesting case of an old lady of 70 years who was operated on for cancer of the breast 25 years ago. After 15 years' complete immunity recurrence took place in the axilla, and five years afterwards numerous cutaneous and subcutaneous growths occupied the arm. Fison warns the profession against assigning any definite limit of recurrence, especially as there is a tendency to contract the time of observation to three years only.

[1] Lettsomian Lectures, Brit. Med. Jour., Mar. 10. [2] Clinical Jour., Jan. 24. [3] Brit. Med. Jour. Jan. 20. [4] Med. Rec., Jan. 20. [5] Med. Rec., Mar. 31. [6] Med. Rec., April 7. [7] Jour. de Médicine de Bordeaux, Feb. 25. [8] Med. Rec., Feb. 24. [9] New York Med. Jour., April 14. [10] Brit. Med. Jour., Mar. 24.

### GENERAL SURGERY.

(*Continued from page* 206.)

**Fractures and Dislocations.**—In his report of a committee on the medico-legal relations of the *x* rays, Professor J. W. White, of Pennsylvania,[8] says that, in fairness to skiagraphy itself as a surgical aid, it should be stated that many of the errors of which examples are cited in the report are avoidable. The habit of taking more than one plate, and of comparing with plates from the normal region corresponding to the injury, will lessen these errors when it is systematically adopted. The report also contains, amongst others, the following summary of its conclusions—viz., that the routine employment of the *x* rays in cases of fracture is not at present of sufficient definite advantage to justify the teaching that it should be used in every case. If the surgeon is in doubt as to his diagnosis, he should make use of this as of every other available means to add to his knowledge of the case, but even then he should not forget the grave possibilities of misinterpretation. There is evidence that in competent hands plates may be made that will fail to reveal the presence of existing fractures, or will appear to show a fracture that does not exist. Likewise, Lucas-Championnière,[9] in a recent discussion at the Société de Chirurgie of Paris, stated that the information afforded by the *x* rays in cases of fracture is unreliable, and also that skiagraphy, applied to such injuries by ignorant and incapable men, may bring trouble on the surgeon. A skiagraph not only presents the mass of callus as being much larger than it really is, but sometimes seems to show considerable thickening of a bone where no callus exists, and where there has not been a fracture. A very slight displacement of the source of light will cause a marked change in the form of the projected part. Thus a supposed deformity due to callus may be readily exaggerated. He, therefore, thinks that skiagraphy, unless carefully and conscientiously practised, is likely to injure the reputation of the practitioner. Dr. D. Walsh[10] concludes an article in The Hospital, on "Recent Progress in Radiography," as follows: "Where a choice is open it is desirable that the Röntgen examination should be placed in the hands of a medical expert. Already the foundations of a flourishing unqualified practice have been laid by handing over the Röntgen examination of patients to instrument makers and electricians. So far as the physician is concerned, it is clear that any report to be of value must come from a Röntgen operator who is versed in medical matters." W. H. Bennett[11] again advocates the use of massage in the treatment of recent fractures. When managed on the line described by him, and alluded to in The Hospital of June 9th, it will be found, if the practitioner will devote the time, and the patient has the patience, that a better result will follow in fractures treated in this way than by any other plan, and that in the end the time which is occupied in the recovery of the patient is little more than half of that which follows the treatment by splints in the ordinary manner. As regards passive movement, he insists that it should always be preceded by smooth-stroking massage, which soothes the irritable muscles so completely that movements of the most complete kind are readily carried out without exciting muscular contraction of a harmful kind. If, on the other hand, passive movement is abruptly attempted without preparing the muscles by previous smooth rubbing, violent muscular action generally occurs, which is not only painful to the patient, but tends to throw the fractured bones out of place. The great advantages of massage are: (1) The ease with which the patient is made comfortable by arresting the muscular spasm, and so relieving the pain; (2) the effecting of rapid absorption of effused blood, &c.; (3) the prevention of stiffness by obviating the formation of adhesions; (4) the prevention of muscle wasting, and the preservation throughout of the normal nutrition of the limb; and (5) the shortening of the time by at least half during which the patient is prevented from resuming the ordinary use of the limb. The only objections to massage are: (1) The frequent difficulty in carrying it out, as, unless a competent masseur or masseuse is available, the time required must be often more than the ordinary practitioner can spare; and (2) the fact that it requires so much intelligence and discretion in the mode of its application may make it difficult at all times to find a person to whom it may be entrusted with safety when the practitioner is not prepared to manage the details himself. A. L. Bennett[12] reports nine cases of perfect union out of 14 cases operated on for ununited fractures, in which Parkhill bone clamp (described in the *Annals of Surgery*, May, 1898) was used. The clamp is easy of adjustment, and when properly applied no motion is possible between the fragments. In operations conducted on strictly aseptic lines the risks of infection are extremely small, and no secondary operation is necessary.

[8] Brit. Med. Jour., May, 19, 1900, p. 1215. [9] Epit. Brit. Med. Jour., May 25, 1900, p. 81; and Bull. et Mém. de la Soc. de Chir., Jan. 2, 1900. [10] The Hospital, Oct. 7, 1899. [11] Lancet, June 2, 1980, p. 1369. [12] Annals of Surgery, March, 1900, p. 327.

### ORTHOPÆDIC SURGERY.

**Forcible Correction of the Deformity of Potts' Disease.**—E. H. Bradford[1] gives an analysis of 610 cases reported by 29 operators. He says: "If the subject be looked at from a pathological point of view, it would seem as if the clinical application of the method would be limited to certain conditions." The curvature in Potts' disease varies very much in its stages of development, and in the case of complete osseous ankylosis resulting from the cured form of the disease the operation is entirely inapplicable. The other stages vary from that of commencement of inflammation with a small carious focus to a large and extensive destruction of bone. If sclerosing or ossifying ostitis is more extensive than destructive ostitis, operative measures become proportionately less desirable than where the destructive ostitis leaves the spine more movable. Bradford is also in accordance with other operators that the presence of an abscess is a contra-indication. He obtains the following results from his analysis: In separately detailed cases, 7 had lasted more than one year, 35 more than six months, 25 more than three months, and 20 less than three months. As the result of the 610 cases, 21 deaths occurred; 5 from meningitis, 4 from general tubercle, 4 from the trauma of the operation, 3 from inter-current disease, and 5 unstated. At the post-mortem examinations all the cases showed a considerable local trauma, and no case showed effectual effort at repair. The immediate dangers appear to be as follows: Respiratory embarrassment in 7 cases, pain in 6 cases, and severe shock in 2 cases. After the operation abscesses were reported in 18 cases. With reference to the important question of paralysis, it was present in 31 cases before the operation, and was relieved by the operation in 17 cases, not relieved in 2, and the result was not stated in 8. Certainly, 7 were distinctly improved. As to the direct effect on the deformity, the nature of the deformity was stated in 229 cases. There was complete correction in 119, incomplete correction in 94, and no gain in 16. The result three months after the operation of 67 cases, in which some gain had been obtained by the operation, was that there was no relapse in 17, some relapse in 44, and complete relapse in 5. Bradford then goes on to remark: "Two questions suggest themselves; the first, how much force is needed in this procedure; and secondly, the best means of applying the force." As to the first question, the amount of force required depends upon what is attempted. If it were desired to break up any ossification, a great deal of force would be necessary, but where the amount of cicatrisation is slight a very slight force suffices, and the less the force the less the danger. Again, the smaller the force used the less the discomfort which the patient suffers. He advocates a plan which has been in use at the Boston Children's Hospital for two years. In this plan little force is used, and that is well under control. The diseased projection is used as the resistant point, and a weight on the trunk on each side of this acts as the straightening force. The appliance by which this correction is done is as follows: An upright which can be raised or lowered by an adjustable screw is furnished with a steel top, having tips so arranged as to steady a zinc plate equipped with holes arranged so as to press at each side of the vertebral spines. If these plates are padded, and placed beneath the patient in such a way that they will lie on each side of the spines at the point of projection, an upward pressure can be exerted by raising the upright by means of a screw attachment. If this be raised to such a height that the head and pelvis hang from a suspended trunk, it is manifest that a strong force is exerted to straighten the spine and correct the curve. This can be increased, if necessary, by a traction force upon the limbs or upon the head and shoulders. The amount of force is regulated by the amount by which the upright is raised.

In cases in which the disease is seated in the upper dorsal region some head support is required. This can be furnished by placing a stiffened leather collar, padded with velvet, round the neck, and including this in a plaster jacket, which passes above the shoulder. When the method of correction is employed, repeated jackets should be applied, and a gradual correction be obtained in preference to the employment of a great deal of force at one sitting. The retention of the corrected spine in its position is a matter of all-importance, for where a cure depends upon a solidified cicatricial ostitis, the tendency to a contraction of cicatrising tissues before ossification has taken place will tend to reproduce the curve, even if thoroughly corrected. Again, as the process of ossification and of cicatrising ostitis is not a rapid one, it is manifest that protection and retention will be required for some time, and the problem of a proper apparatus is not a simple one, as the apparatus must be worn for months, and even years. Bradford favours properly-applied plaster of Paris jackets. With the above conclusions those of Tubby and Jones[2] are very much in accord. These surgeons operated upon 99 cases during three years. Twelve of these cases have died, 15 have discontinued treatment, 44 are walking, some with and some without splints, 28 are recumbent, 17 of the cases developed abscesses, and in many of these the abscesses closed. This compares favourably with 20 per cent. of abscesses in unoperated cases. Twelve have recovered from paraplegia, six are ill with tubercle, and in a large number the curve has been either obliterated or improved. They come to the following conclusions: As to the danger to life the risk is not great, only two cases in 99 having died within a week of the operation, and the death-rate in 25 cases, as compared with 25 controlled cases, was not greater. The operation is a curative measure, so far as paraplegia is concerned, in many cases. The probability of abscess formation is not greater than when the case is left untouched, but is rather less. The dissemination of tubercle is nearly as likely to occur when the spine is left untouched as when it is straightened, but that is almost a negligeable risk. There is no risk of a flail-like spine. Tubby and Jones are of opinion that the operation should not be performed in cervical cases, and not in high dorsal unless paralysis is present. It is not permissible in ankylosed spines, nor in those with very large angular deformities. Tubercle elsewhere is a contra-indication. In children under two it is not wise to perform the operation, as troublesome symptoms, such as vomiting and rise of temperature, are apt to follow, and these children are difficult to keep clean afterwards, and cannot remain quiet in the splints. Adults of over 22 years are not suitable cases. The presence of abscess is a contra-indication unless paraplegia be also coexistent. They think that the operation is suitable in the following cases: In comparatively strong children free from abscess and other forms of tubercle. The treatment should only be undertaken when the spine is yielding. The small, rapidly-forming curves in the mid-dorsal and lumbar regions give the best results. But if large, rapidly-forming curvatures in these regions are treated, they should be done in stages, with a fortnight or month in between. Paraplegia, other things being equal, is a strong indication for the operation. A somewhat extended experience has taught these surgeons that the cases for operation must be selected with great care and discrimination, that it is not an operation to be practised except by those who fully understand the mechanics and pathology of spinal deformities, that personal care of patients must be unremitting, that above everything else the patient should be kept in as good air as possible after operation and during the whole after treatment—the air of large cities being inimical to succcessful ultimate results in this and in other operative procedures undertaken for the treatment of tubercle.

**The Surgical Treatment of Spastic and Paralytic Deformities.**—A. H. Tubby[4] read a paper at a recent meeting of the British Orthopædic Society on this subject. After briefly summarising what is known of the pathology of the various forms of the affection known as infantile spastic paralysis, he thought that in microcephalic, idiotic children surgical operations were contra-indicated, and craniectomy was disastrous. He also did not favour any form of operation if the child was subject to convulsions, but otherwise he rather demurred to the advice often given by physicians and neurologists that nothing should be attempted in these cases. He himself had been accustomed to operate when there had been no convulsions for two or three years, when the mental condition was good, when athetosis was absent, and the case was otherwise favourable for operation. He had not found apparatus to be of any good without operation, as it was unable to fight against the spastic muscles. As an example of the value of surgical procedure in these cases he pointed to talipes equinus, and he said that in his experience the result was a distinct success. In the more severe cases of contraction at the hip and the knees with the pointed foot he was accustomed to divide the flexors and adductors of the thigh as much as they needed, then the hamstrings, and finally the tendo-Achillis. He had also practised a new operation to overcome the deformity of excessive pronation by section of the pronator radii teres. He had succeeded in overcoming the flexion deformity at the elbow and that of the fingers as well.

In the case of severe infantile paralysis much good had been effected by tendon-transplantation, especially in those distressing cases of talipes calcaneus which were not amenable either to shortening of the tendo-Achillis or to instrumental rectification. For talipes calcaneus the best form of operation was to graft the proximal portion of the peroneus longus tendon into the tendo-Achillis, thus at one operation overcoming the calcaneus and the valgus, which so often co-existed in these cases. Indeed, the possibilities of tendon-grafting are almost limitless, and it is found that with massage, galvanism, and proper use of the affected part, the muscle which supplies the power, and is grafted into the paralysed muscle or tendon, undergoes great hypertrophy, and assumes a large part of its functions.

In connection with this subject, an interesting article by Jacob Chesner[4] has been published.

[1] Annals of Surgery, Nov., 1899. [2] Brit. Med. Jour., Mar. 31, 1900.

[3] Brit, Med. Jour, Nov, 18, 1899. [4] Annals of Surgery, Nov., 1899.

